# Contributions and limitations of using machine learning to predict noise-induced hearing loss

**DOI:** 10.1007/s00420-020-01648-w

**Published:** 2021-01-25

**Authors:** Feifan Chen, Zuwei Cao, Emad M. Grais, Fei Zhao

**Affiliations:** 1 Centre for Speech and Language Therapy and Hearing Science, Cardiff School of Sport and Health Sciences, Cardiff Metropolitan University, Cardiff, UK; 2 Center for Rehabilitative Auditory Research, Guizhou Provincial People’s Hospital, Guiyang, China; 3 Department of Hearing and Speech Science, Xinhua College, Sun Yat-Sen University, Guangzhou, China

**Keywords:** Noise-induced hearing loss, Machine learning, Prediction models, Discrimination risk

## Abstract

**Purpose:**

Noise-induced hearing loss (NIHL) is a global issue that impacts people’s life and health. The current review aims to clarify the contributions and limitations of applying machine learning (ML) to predict NIHL by analyzing the performance of different ML techniques and the procedure of model construction.

**Methods:**

The authors searched PubMed, EMBASE and Scopus on November 26, 2020.

**Results:**

Eight studies were recruited in the current review following defined inclusion and exclusion criteria. Sample size in the selected studies ranged between 150 and 10,567. The most popular models were artificial neural networks (*n* = 4), random forests (*n* = 3) and support vector machines (*n* = 3). Features mostly correlated with NIHL and used in the models were: age (*n* = 6), duration of noise exposure (*n* = 5) and noise exposure level (*n* = 4). Five included studies used either split-sample validation (*n* = 3) or ten-fold cross-validation (*n* = 2). Assessment of accuracy ranged in value from 75.3% to 99% with a low prediction error/root-mean-square error in 3 studies. Only 2 studies measured discrimination risk using the receiver operating characteristic (ROC) curve and/or the area under ROC curve.

**Conclusion:**

In spite of high accuracy and low prediction error of machine learning models, some improvement can be expected from larger sample sizes, multiple algorithm use, completed reports of model construction and the sufficient evaluation of calibration and discrimination risk.

## Introduction

Noise can be defined as an unwanted sound that may cause unpleasant, annoyance, and distraction. Excessive noise exposure has been shown to have a range of detrimental effects on people’s hearing as well as their general health and psychological well-being (e.g., stress, anxiety and insomnia) (Sayler et al. 2019; Williams et al. 2015; Zare et al. 2018). Noise-induced hearing loss (NIHL) is often described as a hearing loss caused by exposure to sound at significant intensity over an extended period of time (Abdollahi et al. 2018; Jansen et al. 2009; South 2013). It is considered one of the most important and avoidable occupational health issues throughout the world, due to its negative influence on communication, cognitive function and psychological status (Basner et al. 2014; Deafness and Hearing 1998; Fligor and Cox 2004; Meyer-Bisch 1996; Opperman et al. 2006). Prevalence of NIHL ranges from 7 to 21% among workers in different countries (Nelson et al. 2005) and it is the second most common type of hearing loss following presbycusis (Imam and Hannan 2017). Excessive exposure to loud noise leads to permanent damage within the Organ of Corti structures and an elevation of hearing thresholds (Hirose and Liberman 2003). The typical feature seen in early stage NIHL is the audiometric “notch” at frequencies between 3 and 6 kHz seen in pure tone audiometry (Rabinowitz et al. 2006). With continuous noise exposure over a long period of time, the degree of hearing loss and affected frequency range increases (Lie et al. 2015).

Currently many countries have a permissible exposure limit of 85 dBA with the 3-dB exchange rate and use this as the formula to calculate an individual’s daily noise dose and duration, i.e. the recommended maximum (or 100%) daily noise dose over an eight-hour period should not exceed an average of 85 dBA (Arenas and Suter 2014). Consequently, a noise exposure over 85 dBA for longer than 8 h a day over a long period of time has been generally considered as the most important risk factor for NIHL (Korver et al. 2017; Tikka et al. 2017).

High level of noise exposure may initially give rise to a temporary threshold shift (TTS), which could recover after a few hours, days or weeks with removal of the noise source (Ryan et al. 2016). However, a longer duration and cumulative noise exposure will lead the TTS to a permanent threshold shift (PTS), damaging hair cells and cochlear nerve irreversibly with a resultant noise-induced hearing loss (Liberman 2016). In addition, impulse sounds have an extremely high sound pressure level within a very short duration and can induce cochlear injury at higher frequencies (> 3 kHz) (Lie et al. 2016).

Apart from the type and intensity of noise and duration of exposure, other factors can influence the occurrence of NIHL. Demographic, genetic, behavioural (e.g. hearing protection device usage) factors as well as general health condition can all affect an individual’s susceptibility to work-related sound exposure (Bovo et al. 2007; Kähäri et al. 2001). As examples, Konings et al. (2007) identified a significantly higher occurrence of NIHL among workers possessing a mutation of the catalase gene responsible for management of cellular oxidative stress. Wong et al. (2013) identified an increased incidence of NIHL in workers who possess mutations to genes that alter the K^+^ concentration in endolymph, such as KCNE1 and KCNQ4. These disturb the normal function of mechano-transduction channels in hair cells. These genetic factors were significantly correlated with NIHL occurrence (Pawelczyk et al. 2009; Van Laer et al. 2006).

Roberts et al. (2018) compared the predictability of NIHL risk using two noise measurement criteria: average noise level and equivalent continuous average. They found that equivalent continuous average performed better especially in predicting hearing thresholds at 0.5, 3 and 4 kHz. However, it is problematic to predict NIHL using only exposure variables and without considering other important factors indicated above. It is important to identify different risk factors and their interactions to more accurately predict the probability of NIHL occurrence.

Machine learning (ML) has been widely applied to automatically identify inter-correlations between data that would normally require a great deal of manpower and be difficult to define manually (McKearney and MacKinnon 2019). The application of ML to the field of Audiology has shown promise, because of its effectiveness in analyzing non-linear relationships between data such as predicting hearing thresholds of patients who are exposed to specific risk factors (Chang et al. 2019). Abdollahi et al. (2018) constructed eight ML models to predict sensorineural hearing loss (SNHL) after chemoradiotherapy, of which five had over 70% accuracies and precisions. Other studies showed similar high accuracies with ML models used to predict sudden sensorineural hearing loss (SSNHL) and otoxic-induced hearing loss (Bing et al. 2018; Tomiazzi et al. 2019). Varied accuracies between 64 and 99% were reported by different studies using different ML algorithms and inputs to predict risk factors for NIHL (Aliabadi et al. 2015; Farhadian et al. 2015; Kim et al. 2011; Mohd Nawi et al. 2011; Zhao et al. 2019a).

It is noteworthy, however, that not all ML algorithms are substantially superior to traditional statistical regression analysis in terms of model performance when predicting hearing loss caused by specific risk factors (Abdollahi et al. 2018; Bing et al. 2018; Farhadian et al. 2015). To the best of our knowledge, there is no literature review evaluating the quality of ML models to predict NIHL. Currently, the benefits and challenges of applying ML algorithms to predict NIHL remain unclear. The present review aimed to clarify the contributions and limitations of applying machine learning tools to predict NIHL by analyzing ML model performance and the procedure of model construction. The significant outcomes would contribute towards a better understanding of ML tools to predict the susceptibility to NIHL and thus facilitate its prevention.

## Methods

### Search strategy

To identify studies related to the application of ML to prediction of NIHL, we executed a literature search in PubMed, EMBASE and Scopus on November 26, 2020. Search terms were designed to cover all possible papers: (algorithm OR artificial intelligence OR data mining OR machine learning OR neural network OR deep learning OR decision tree OR random forest OR multilayer perceptron OR support vector machine OR classification tree) AND (noise OR noise induced OR noise exposure) AND (hearing loss OR hearing impairment OR hearing problem OR hearing disease OR threshold shift).

### Literature selection

The first two authors (F.C and Z.C) screened the title and abstract of the searched papers independently. Subsequently, the same two confirmed the full text of selected papers and evaluated their eligibility. The 22-item TRIPOD checklist published by Moons et al. in 2015 was used to evaluate the quality of the study design, model development and validity of ML algorithms applied to medical diagnosis or prognosis prediction. As a result, journal articles published after 2015 were recruited as one of the inclusion criteria. Other important inclusion criteria were also clearly defined and classified. Table 1 summarizes the key components of the inclusion and exclusion criteria.

**Table 1:** Inclusion and exclusion criteria for search strategy

	Detailed items
Inclusion criteria	Published: within 5 years, in English Participants: adults with noise induced hearing loss, had long-term working experience in the noise environment; no history of ear surgery, severe brain injury, tumors or ototoxic drug use, no diabetes mellitus Study design: Clinical trials Outcome measure: different machine learning algorithms such as artificial neural network, random forest, support vector machine
Exclusion criteria	Study design: reviews, case reports/series, meta-analyses, animal studies Study objective: studies investigating genetics, cytology, assistive hearing devices, audiological assessment

### Data extraction and analysis

To systematically appraise the included studies, CHARMS guideline was used to review the performance of ML models as proposed by Moons et al. (2014) for critical appraisal and data extraction for reviews related to machine learning. General information was collected by the first two authors, including study aim, study and model design, input, output and main results (Table 2). The performance of the ML algorithms was evaluated on the basis of: accuracy, precision, receiver operating characteristics (ROC) curve, area under the curve (AUC), prediction error/root-mean-square error (RMSE), sensitivity and specificity. The procedure used by the different algorithms in predicting or classifying NIHL was summarized and critically analyzed as well ( Table 3). Analysis included input selection tools, algorithms, calibration performance, discrimination performance, validation tool, strength and weakness.

**Table 2:** General information of the included Studies

Study	Study aim	Study and model design	Input	Output	Results
Farhadian et al. (2015)	To analyze the potential of artificial neural networks and logistic regression techniques for estimation of hearing impairment among industrial workers	Sample size (*n*) 210 M: F 210:0 Age range 35.5 ± 4.6 Algorithm ANN	Categorical Smoking (yes or no), Using HPD usage (continuous, intermittent or no) Continuous Age, exposure duration, noise exposure level	Categorical 74.2% < 25 dB 23.4% 25–40 dB 2.4% 41–60 dB (WHO) Continuous N/A	ANN performed better than LR either in the train phase (accuracy: 91.4% vs 87.85%) or the test phase (accuracy: 88.6% vs 84.28%) Cohen’s kappas of ANN were 81and 66.3 in the train and test group, which outperformed than LR (72.7 and 51.3) ROC curves showed better performance of ANN to predict the grades of hearing loss than that of the logistic regression model
Aliabadi et al. (2015)	To present an empirical model for the prediction of the hearing loss threshold among noise-exposed workers	Sample size (*n*) 210, M: F 210:0 Age range 35.5 ± 4.6 Algorithm ANN	Categorical Smoking (yes or no), Using HPD usage (continuous, intermittent or no) Continuous Age, exposure duration, noise exposure level	Categorical N/A Continuous 24.8 ± 7.32 dB	The RMSE (dB) of ANN was lower than multiple linear regression (train phase: 2.40 vs 4.04, test phase: 2.60 vs 4.47) *R*^*2*^ of ANN was higher than the regression model in both phrases (train phase: 0.88 vs 0.69, test phase: 0.89 vs 0.67) ANN model was able to predict the hearing thresholds of three workers with the difference below 1.5 dB
Greenwell et al. (2018)	To analyze historical hearing sensitivity data to determine factors associated with an occupationally significant change in hearing sensitivity in U.S. Air Force aviation-related personnel	Sample size (*n*) 10,567 M: F 9,589:978 Age range: 24.5 ± 4.24 Algorithm RF	Categorical Gender, AFSC Continuous Age, time interval between each audiogram (yrs)	Categorical STS category: 8332 < 10 dB 2215 ≥ 10 dB (OSHA) H1 category: 1109 non-H1 9438 H1 (OSHA) Continuous N/A	The RF model accounted for approximately 20% prediction error of predicting STS and non-H1 profile It reached around 80% accuracy in both subgroups The included variables in the RF model could explain 16.8% of the change in hearing threshold change
Zare et al. (2019)	To use the C5 algorithm to determine the weight of factors affecting the workers’ hearing loss based on the audiometric data	Sample size (*n)* 150 M: F N/A Age range: G1: 37.66 ± 9.91 G2: 35.56 ± 11.45 G3: 41.76 ± 10.93 Algorithm C5 algorithm	Categorical Age, working experience (yrs) Continuous 250 Hz, 500 Hz, 1 kHz, 2 kHz, 4 kHz, 8 kHz	Categorical 106 < 25 dB 35 25–40 dB 7 41–60 dB 2 61–80 dB (WHO) Continuous N/A	The accuracy of C5 was 99.33%
Zhao et al. (2019a)	To demonstrate the feasibility of developing machine learning models for the prediction of hearing impairment in humans exposed to complex non-Gaussian industrial noise	Sample size (*n*) 1,113 M: F 802: 311 Age range 30–50 Algorithm RF, Adaboost model, MLP, SVM	Categorical N/A Continuous Age, duration of noise exposure, LAeq of the noise, median kurtosis of the noise	Categorical 892 < 25 dB 221 ≥ 25 dB (NIOSH) Continuous Hearing thresholds (Mean ± SD)	SVM model had the highest accuracy of predicting hearing impairment (0.8014) but was not significantly different (*p* > 0.01) from the other three (AdaBoost: 0.7862; MLP: 0.7898; RF: 0.7970) SVM performed highest AUC to predict hearing impairment (0.808), compared with MLP (0.711), RF (0.663) and Adaboost (0.661) ROC curves of four algorithms were not significantly different, with SVM outperformed MLP reached lowest RMSE value (2.727) to predict the hearing thresholds of workers in all except three factories with the difference less than 2.5 dB HL
Zhao et al. (2019b)	To predict hearing impairment in workers exposed to both Gaussian (G) and non-Gaussian (non-G) industrial noises	Sample size (*n*) 2,110 M: F 1,530:580 Age range 35.8 ± 10.1 Algorithm RF, SVM	Categorical N/A Continuous Age, L_Feq_, L_Aeq,_ L_eq_500_, L_eq_1000_, L_eq_2000_, L_eq_4000_, exposure duration, mean kurtosis	Categorical recommendation: 1437 < 25 dB 673 ≥ 25 dB (NIOSH) Continuous N/A	The accuracy of RF was highest (> 75%) when the number of included inputs was 9 The highest accuracy of 75.3% was reached by SVM, compared with 68.6% from ISO-1999 model The precision (74.3% vs 71.0%), recall (68.9% vs 51.0%) and *F1* score (71.5% vs 59.4%) of SVM in all groups were significantly better than the ISO-1999 model
ElahiShirvan et al. (2020)	To utilize audiometric data to weigh and prioritize the factors affecting workers’ hearing loss based using the SVM algorithm	Sample size (*n*) 150 M: F N/A Age range: G1: 37.66 ± 9.91 G2: 35.56 ± 11.45 G3: 41.76 ± 10.93 Algorithm SVM	Categorical Age, working experience (yrs) Continuous 250 Hz, 500 Hz, 1 kHz, 2 kHz, 4 kHz, 8 kHz	Categorical 106 < 25 dB 35 25–40 dB 7 41–60 dB 2 61–80 dB (WHO) Continuous N/A	The accuracy of SVM model was 94% The model predicted 25.71% participants with mild hearing loss as normal hearing
Zare et al. (2020)	To model the significance of a variety of factors influencing the development of hearing loss among industry workers by using a neural network algorithm	Sample Size (n) 150 M: F N/A Age range: G1: 37.66 ± 9.91 G2: 35.56 ± 11.45 G3: 41.76 ± 10.93 Algorithm ANN	Categorical Age, working experience (yrs) Continuous 250 Hz, 500 Hz, 1 kHz, 2 kHz, 4 kHz, 8 kHz	Categorical 106 < 25 dB 35 25–40 dB 7 41–60 dB 2 61–80 dB (WHO) Continuous N/A	The accuracy of neural network model was 99.3% when it predicted the hearing loss of all participants The lowest accuracy of prediction was 80% in the group exposed to over 87 dBA noise level, with the accuracy over 98% in the other groups

*ANN* artificial neural networks, *HPD* hearing protective devices, *WHO* World Health Organization, *LR* logistic regression, *ROC* receiver operating characteristics, *RMSE* root-mean-square error, *RF* random forest, *AFSC* Air Force Specialty Code, *STS* significant threshold shift (STS if hearing thresholds within 2–4 kHz is ≥ 10 dB HL in at least one ear), *H1* non-H1 if hearing threshold is > 25 dB HL at 500, 1000, or 2000 Hz, > 35 dB HL at 3000 Hz or > 45 dB HL at 4000 or 6000 Hz, *OSHA* occupational safety and health administration, *MLP* multilayer perceptron, *SVM* support vector machine, *NIOSH* national institute for occupational safety and health, *AUC* area under the ROC curve, *L*_*Aeq*_ equivalent A-weighted SPL, *L*_*Aeq*_ equivalent F-weighted SPL, *L*_*eq*_ equivalent continuous sound level

**Table 3:** Strength and weakness of the procedure of different ML algorithms to predict or classify NIHL

Study	Model construction procedure		Strength	Weakness
Farhadian et al. (2015)	Input selection tools	Correlation matrix	Evaluated the candidacy of variables Evaluated calibration performance by multiple metrics Specified the validation method	Single algorithm usage The performance of ANN with different numbers of neurons in the training phase was not informed
	Algorithms	ANN		
	Calibration performance	Accuracy, Cohen's kappa coefficient		
	Discrimination performance	ROC		
	Validation tool	Split-sample validation		
Aliabadi et al. (2015)	Input selection tools	Correlation matrix	Evaluated the candidacy of variables Evaluated calibration performance by multiple metrics Specified the validation method	Single algorithm usage Not informed the performance of different numbers of neurons in the training phase Not evaluated discriminative risk Only used 10% of the data for validation
	Algorithms	ANN		
	Calibration performance	RMSE, *R*^*2*^		
	Discrimination performance	N/A		
	Validation tool	Split-sample validation		
Greenwell et al. (2018)	Input selection tools	N/A	Evaluated the candidacy of variables Evaluated calibration performance by multiple metrics Specified the validation method	Single algorithm usage No evaluated discriminative risk
	Algorithms	RF		
	Calibration performance	Accuracy, prediction error, *R*^*2*^		
	Discrimination performance	N/A		
	Validation tool	Split-sample validation		
Zare et al. (2019)	Input selection tools	N/A	Evaluated calibration performance by accuracy	Not evaluated the candidacy of variables Single algorithm usage Not evaluated discriminative risk No validation tools
	Algorithms	C5 algorithm		
	Calibration performance	Accuracy		
	Discrimination performance	N/A		
	Validation tool	N/A		
Zhao et al. (2019a)	Input selection tools	t-test	Evaluated the candidacy of variables Multiple algorithm usage Evaluated calibration performance by multiple metrics Specified the validation method	N/A
	Algorithms	RF, Adaboost model, MLP, SVM		
	Calibration performance	Accuracy, RMSE		
	Discrimination performance	ROC, AUC		
	Validation tool	tenfold cross-validation		
Zhao et al. (2019b)	Input selection tools	RF	Evaluated the candidacy of variables Evaluated calibration performance by multiple metrics Specified the validation method	Single algorithm usage Not evaluated discriminative risk
	Algorithms	SVM		
	Calibration performance	Accuracy, precision, recall, *F1* score		
	Discrimination performance	N/A		
	Validation tool	tenfold cross-validation		
ElahiShirvan et al. (2020)	Input selection tools	N/A	Evaluated calibration performance by accuracy	Not evaluated the candidacy of variables Single algorithm usage Not evaluated discriminative risk No validation tools
	Algorithms	SVM		
	Calibration performance	Accuracy		
	Discrimination performance	N/A		
	Validation tool	N/A		
Zare et al. (2020)	Input selection tools	N/A	Evaluated calibration performance by accuracy	Not evaluated the candidacy of variables Single algorithm usage Not evaluated discriminative risk No validation tools
	Algorithms	ANN		
	Calibration performance	Accuracy		
	Discrimination performance	N/A		
	Validation tool	N/A		

*NIHL* noise-induced hearing loss, *ANN* artificial neural networks, *ROC* receiver operating characteristics, *RMSE* root-mean-square error, *RF* random forest, *MLP* multilayer perceptron, *SVM* support vector machine, *AUC* area under the ROC curve

## Results

### General characteristics of the include studies

**Fig. 1:**
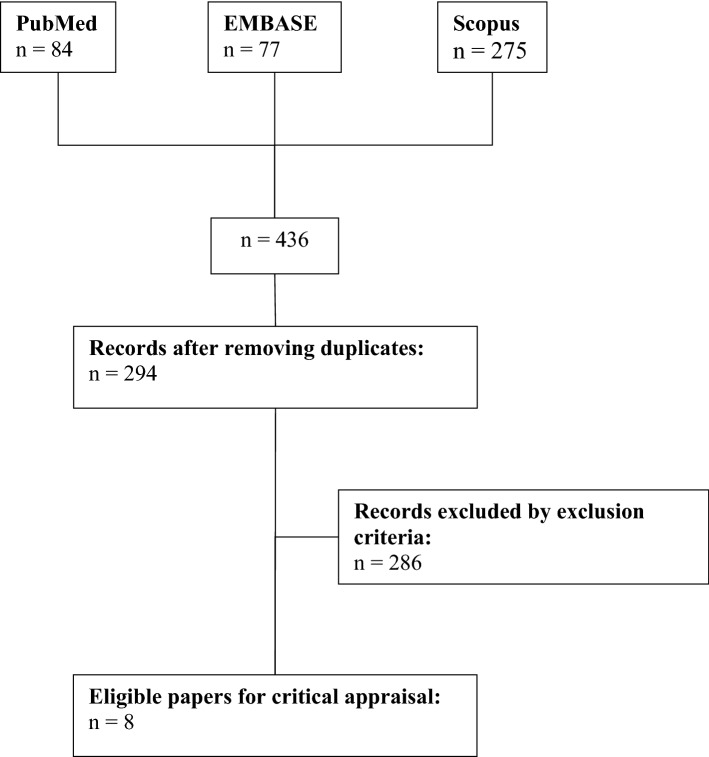
Flow diagram of the search strategy for studies assessing the predictability of machine learning models for NIHL

The current search strategy identified 436 papers based on the inclusion criteria with 294 left after the removal of duplicates. We removed 286 records according to the exclusion criteria or considered as irrelevant. Finally, eight eligible papers were included in the current review ( Fig. 1).

Table 2 summarizes the specific characteristics of the studies. The number of participants ranged from 150 to 10,567 (median: 210). Seven studies recruited participants with ages ranging from 30 to 50. Only one study recruited significantly younger participants with all participants recruited from the U.S. Air Force (Greenwell et al. 2018). As shown in Table 2, the gender distribution was imbalanced with many more males than females in five studies (total male vs. female: 12,341 vs. 1869) (Aliabadi et al. 2015; Farhadian et al. 2015; Greenwell et al. 2018; Zhao et al. 2019a, b). There was no information of gender in three included studies (ElahiShirvan et al. 2020; Zare et al. 2019, 2020).

Different categorization approaches and grading systems of NIHL were used in the studies, e.g. 25 dB HL as a criterion defined by the National Institute for Occupational Safety and Health (NIOSH) (Zhao et al. 2019a, b), and the grade systems recommended by the World Health Organization (WHO) and Occupational Safety and Health Administration (OSHA) (ElahiShirvan et al. 2020; Farhadian et al. 2015; Greenwell et al. 2018; Zare et al. 2019, 2020). By contrast, the averaged hearing thresholds of participants were also used as a variable in two studies (Aliabadi et al. 2015; Zhao et al. 2019a). Of these studies, Zhao et al. (2019a) reported the overall better performance (AUC and accuracy) of four algorithms to predict noise-induced hearing impairment defined by OSHA in comparison to using NIOSH’s definition. It should be noted that the different approaches to defining NIHL are very likely to influence the outcomes of the ML models.

A number of variables were considered as potential risk factors for NIHL including: age, gender, duration of noise exposure, noise exposure level, smoking habit, HPD use, time interval between each audiometric measurement (in years), median kurtosis of the noise, individual hearing thresholds at the frequencies of 0.25, 0.5, 1, 2, 4 and 8 kHz, equivalent continuous sound level at 0.5, 1, 2 and 4 kHz (L_eq_500/1000/2000/4000_) and Air Force Specialty Code (AFSCs). Of these, age (*n* = 8), duration of noise exposure (*n* = 7) and noise exposure level (*n* = 6) were the parameters mostly correlated with NIHL, followed by other predictive factors, such as median kurtosis of noise (*n* = 2), L_eq_1000_ (*n* = 1) and L_eq_500_ (*n* = 1). Although age and exposure duration were both highly correlated with hearing thresholds, these two variables may underestimate the risk of multicollinearity, which would undermine the validity of their predictive effect to the outcome (Alin 2010). In addition, the hearing threshold at 4 kHz was found as an effective predictor for overall NIHL (ElahiShirvan et al. 2020; Zare et al. 2019, 2020). This result is in keeping with the hypothesis that high frequencies are most vulnerable after excessive noise exposure (Rabinowitz 2000). By contrast, the studies showed the lesser contribution of categorical data to predict the occurrence of NIHL, such as gender, hearing protection device (HPD) usage (defined as ‘continuous’, ‘intermittent’ or ‘no’), and smoking status (‘yes’ or ‘no’). Therefore when converting numeric data into a categorical variable (e.g. convert HPD usage duration to usage patterns) in ML to predict NIHL, it may cause selection bias due to less information reflecting the relationship between a variable and the output(s) (Altman and Royston 2006). For instance, two studies merged workers who used HPD 2 h per day and those who used 6 h per days into the same group defined as ‘continuous’ HPD group (Aliabadi et al. 2015; Farhadian et al. 2015). As a result, the relationship between the protective effect of using HPD and the duration of usage could be potentially neglected. In addition, the influence of noise type on the occurrence of NIHL in this study is unclear (Greenwell et al. 2018). Although there was a higher occurrence of NIHL in participants who were exposed to higher level of noise classified on the basis of AFSCs, the study did not evaluate the nature of environmental noise every group of participants with different AFSC experienced.

Several ML models were used in the included studies, i.e. random forest (RF), artificial neural networks (ANN) or multilayer perceptron (MLP), support vector machine (SVM), C5 algorithm and AdaBoost model. Three studies used more than two different machine learning algorithms, though one study applied RF to determine qualified inputs only and did not compare the predictive performance with another model (Zhao et al. 2019b). The most commonly used ML model was ANN (including one using MLP) in four studies, followed by RF and SVM in three studies (Table 2). Only 2 studies contained case analysis to evaluate the practical performance of ML models (Aliabadi et al. 2015; Zhao et al. 2019a). Three studies contained different regression models including logistic regression (LR), multiple linear regression (MLR) and the linear mixed-effects model (LMM) (Aliabadi et al. 2015; Farhadian et al. 2015; Greenwell et al. 2018).

### Critical appraisal of model constructions

Table 3 summarizes important information regarding the model construction as well as the strength and weakness of individual studies. Four included studies performed the analysis using specific tools by selecting different inputs and all studies defined the variables clearly. However, only one measurement used to evaluate calibration performance or lacking discrimination evaluation in the included studies made the full appraisal of the ML algorithms difficult. Two studies presented ten-fold cross-validation, whereas three applied split-sample validation.

Evaluating the candidacy of inputs for predictive power helps to prevent overfitting, which refers to the circumstances where a model is tailored too much by data to generalize to new data sets (Lever et al. 2016). Only four studies used multivariable models (e.g. correlation matrix, RF) to select inputs, but only two performed the statistical analysis to define those inputs which were significantly correlated with the target variable (Zhao et al. 2019a, b). None of the studies utilized a separate dataset to conduct feature selection, which is imperative to prevent predictor selection bias especially for regression models (Singhi and Liu 2006).

Although various algorithms were chosen and used in the included studies, only one study performed multiple models to compare the predictive performance of NIHL (Zhao et al. 2019a). The remaining studies did not compare with other machine learning classifiers especially with recent machine learning approaches, such as deep learning. In addition, although two studies evaluated the performance of ANN with different structures and found ANN with one hidden layer and ten neurons to be superior, the results with different numbers of neurons were not informed (Aliabadi et al. 2015; Farhadian et al. 2015).

According to the CHARMS guideline, compulsory model performance measures should at least consist of calibration and discrimination (Moons et al. 2014). Calibration refers to the comparison between predicted and observed results, whilst discrimination represents the degree of distinguishing those at higher risk of having an event from those at lower risk (Alba et al. 2017). Calibration usually comprises accuracy, precision, *R*^*2*^ or F_1_ score (Siblini et al. 2020). Discrimination risk could be assessed by the ROC and the AUC (Moons et al. 2014). All studies clarified the component metrics to evaluate performance except the study by Greenwell et al. (2018) which performed accuracy, prediction error and *R*^*2*^ without any definition. Various calibration measures were applied including accuracy (*n* = 8), RMSE/prediction error (*n* = 3), precision (*n* = 1), recall (*n* = 1), F_1_ score (*n* = 1) and/or *R*^*2*^ (*n* = 1). The discrimination risk, however, was only evaluated in two studies by the ROC curve and/or the AUC (Farhadian et al. 2015; Zhao et al. 2019a).

In regard to model validation, either split-sample validation (Aliabadi et al. 2015; Farhadian et al. 2015; Greenwell et al. 2018), or ten-fold cross-validation (Zhao et al. 2019a, b) was applied. Slip-sample validation randomly divides samples into a training group and a validation group (Moons et al. 2014). N-fold cross-validation is a procedure to prevent performance bias of models in which the total sample is divided into N groups and each Nth group tests the model in fold N sequentially, while N-1 groups are applied to train the model (Mellor et al. 2018). However, it should be noted that split validation rather than N-fold cross-validation was proved to produce unbiased performance with limited sample size (*n* < 1,000) (Vabalas et al. 2019). It is noteworthy that Aliabadi et al. (2015) used 10% of the data for validation, but the small sample size (*n* = 210) may cause the risk of overfitting. In addition, three included studies that shared the same database did not apply any validation tool, which may have the high risk of overfitting (ElahiShirvan et al. 2020; Zare et al. 2019, 2020).

### Critical appraisal of the performance of ML algorithms: contributions and limitations

Although the performance of different prediction models varied in the 8 studies, accuracies of included algorithms were relatively high and the prediction errors (or RMSE) consistently outperformed regression models. On the other hand, apart from the limited number of results, the sensitivities and specificities of some ML models informed by the ROC curves were not favorable and the results of the AUC were relatively low in two of four models in one study (Zhao et al. 2019a) (Table 2).

Accuracy refers to the ratio of correctly classified samples in the total sample (Tharwat 2018). In the present review, the averaged accuracy of various ML algorithms was greater than 75%. Of these, the accuracies obtained from RF models ranged from 75.5% to 80% in three studies (Greenwell et al. 2018; Zhao et al. 2019a, b) and there were accuracies of 75.3–94% when using SVM models (ElahiShirvan et al. 2020; Zhao et al. 2019a, b). It reached 78.98% and 78.62% by MLP model and AdaBoost, respectively (Zhao et al. 2019a). The accuracy over 99% was achieved using C5 algorithm in the study by Zare et al. (2019), but the potential overfitting problem and the issue of small sample size (*n* = 150) should be noted. Although Farhadian et al. (2015) and Zare et al. (2020) reported the accuracy over 88% using ANN model, the small sample size in both studies could also lead to overfitting problem and be difficult for generalization, and consequently resulted in spuriously high accuracy (Vabalas et al. 2019). By contrast, three of five studies contained different regression models including LR, MLR and LMM (Aliabadi et al. 2015; Farhadian et al. 2015; Greenwell et al. 2018). The accuracy of LR did not differ significantly from the result obtained from ANN model (LR vs ANN: training group: 87.85% vs 91.4%, test group: 84.28% vs 88.6%) in both training and test group (Farhadian et al. 2015). There was no comparison of the accuracy between ANN and MLR (Aliabadi et al. 2015) or SVM and LMM (Greenwell et al. 2018).

The prediction error and RMSE are used to measure the difference between the predicted values, where lower values reflect higher accuracy of the prediction models (Hyndman and Koehler 2006). The overall RMSEs of five algorithms were below 3 dB. In particular, ANN achieved the lowest RMSE of 2.4 and 2.6 in the training and test group (Aliabadi et al. 2015). One study found MLP RMSE of 2.727 in predicting hearing thresholds, RF was 2.858, as well as Adaboost (2.894) and SVM (2.942) (Zhao et al. 2019a). By contrast, MLR showed over 4 dB RMSE in both training and test group, which was significantly higher than ANN (Aliabadi et al. 2015). For RF model, it obtained an approximate 20% prediction error, which was not satisfactory (Greenwell et al. 2018). Limited inputs (*n* = 4) in this study might misguide the algorithms and return the poor result.

In terms of case analysis, Aliabadi et al. (2015) showed that ANN model was able to predict three workers’ hearing thresholds with a difference below 1.5 dB HL. In addition, by MLP model, the difference between the measured and predicted hearing thresholds was less than 2.5 dB HL in another study (Zhao et al. 2019a). However, neither of them applied separated datasets to prevent the potential overfitting problem.

The ROC curve represents the tradeoff between true positive rate (sensitivity) and false positive rate (1-specificity), which measures the discriminative ability of ML models (Tharwat 2018). Although 100% sensitivity and approximately 90% specificity were achieved by ANN (Farhadian et al. 2015), the performances of 4 models based on the ROC curves in another study were limited (Zhao et al. 2019a). More specifically, SVM was the best but still could not balance the sensitivity and specificity over 75% simultaneously and the performance of the other three (RF, MLP and Adaboost) were significantly inferior with no more than 65% simultaneously. On the other hand, despite no result of the ROC curve, Zhao et al. (2019b) reported only 68.9% sensitivity of SVM. Biased inputs which merely included noise-related features might render it difficult to predict hearing impairment. Similar to regression model, the ROC curve of LR presented limited outcomes with highest sensitivity at 90% and no more than 70% specificity (Farhadian et al. 2015). Moreover, the AUC is also used to compare the performance of different models in the ROC curves, ranging from 0 to 1 (Tharwat 2018). The higher values a model obtains, better ROC performance it represents. In the study by Zhao et al. (2019a), the result of SVM (0.808) was significantly higher than MLP (0.711), RF (0.663) and Adaboost (0.664), suggesting a better discriminative power of the SVM model in predicting NIHL. Notably, only this study evaluated the ROC performance, and thus the outperformance of SVM remained putative.

## Discussion

### Contribution of ML models to predict NIHL

The present review demonstrates that ML models had higher accuracies and lower prediction errors when compared to regression models. The majority of included studies used accuracy to evaluate various ML models with the predictive ability for NIHL ranging from 75.3% to 99% due to the heterogeneity of datasets and model development. In particular, three studies with the accuracy over 90% provided limited information on input selection and no validation tools were applied (ElahiShirvan et al. 2020; Zare et al. 2019, 2020). Two previous studies which did not meet the inclusion criteria demonstrated similar issues. Although the study by Mohd Nawi et al. (2011) reported over 99% accuracy of the prediction model, incomplete information of the model construction created the risk of bias. Another study constructed a large database (*n* = 2,420,330) to analyzed the impact of diverse noise to the generation of NIHL using ANN but unraveled the unsatisfactory performance with less than 65% accuracy, which was no better than LR model (Kim et al. 2011). The accuracies of some algorithms were also investigated in several studies which either tried to predict hearing loss with specific etiologies, such as sudden hearing loss (Bing et al. 2018; Park et al. 2020), ototoxic hearing loss (Tomiazzi et al. 2019) and cochlear dead regions (Chang et al. 2019), or predict SNHL by specific auditory measures, such as OAE (de Waal et al. 2002; Liu et al. 2020; Ziavra et al. 2004) and ABR (Acır et al. 2006; Molina et al. 2016). Similarly, five studies did not evaluate or describe the significance of input to cochlear dead regions (Chang et al. 2019; de Waal et al. 2002; Liu et al. 2020; Tomiazzi et al. 2019; Ziavra et al. 2004). Therefore, the validity of the accuracy metric is highly dependent on the transparency of model development and input selection.

Notably, it is not appropriate to determine if an algorithm has a favorable performance simply by accuracy as this measure might be biased if the distribution of data is imbalanced, in which situation some classes are more frequent in comparison with others (Krawczyk 2016). Consequently, those models perform biased and conservative to predict the minority class still are able to reach the high accuracy. In this case, precision, recall, F_1_ score, prediction error and the ROC/AUC curve should be applied to evaluate the predictability of algorithms to prevent an overestimation of predictive power (Fabris et al. 2017).

### Model selection to predict NIHL

Based on the characteristics of study design, supervised machine learning is more suitable to construct predictive models. In general, the procedure of supervised ML usually consists of data collection, inputs extraction and selection, algorithms selection, training, testing and validation (Kuncheva 2014). Supervised algorithms predict or classify labelled output (i.e., NIHL) by discovering relationships between features in the training group, aiming to find relationships and patterns in the data that might be too complex to visualize manually (Fabris et al. 2017; Low et al. 2020).

With regard to the application of ML models, RF, ANN (including MLP) and SVM were the most frequently used models in the included studies, which achieved favorable performance. In addition, different studies also applied more than one model to predict other types of hearing loss or hearing-related problems. Three types of algorithms achieved good performance in predicting sudden hearing loss (Bing et al. 2018; Park et al. 2020), ototoxic hearing loss (Tomiazzi et al. 2019) and/or SNHL with specific risk factors (Chang et al. 2019). Also, several studies successfully applied ANN or SVM to clarify different types of SNHL based on the morphology of OAE (de Waal et al. 2002; Liu et al. 2020; Ziavra et al. 2004) or ABR (Acır et al. 2006).

Although various ML models outperformed regression models based on the results of current review, Christodoulou et al. (2019) discovered no difference between two types of models in 71 clinical prediction studies. However, due to the heterogeneous methodologies and aims, they only analyzed the AUC without further delineating the difference of performance between ML and LR in analyzing different prediction problems. Because ML models are trained to learn from data, the sample size should be sufficiently large to contain variety and patterns and thus minimize the errors and bias that are inherent in the procedure of data collection (Mellor et al. 2018).

By contrast, traditional regression models rely on assumptions and known information between data so that require smaller sample sizes to discover relationships, which performs better in terms of interpreting the relationship between different variables (Zhang et al. 2016). Several previous studies used statistical analysis to explore etiological factors of NIHL. A multiple linear regression model revealed that TTS at 4 kHz was one of the significant predictive factors for a PTS of the average thresholds from 2 to 4 kHz (equal to NIHL). Using 14 dB TTS as the cutting point could achieve good sensitivity (82%), though specificity (53%) was relatively poor (Moshammer et al. 2015). The other multiple linear regression model was developed by Xie et al. (2016) using age and cumulative noise exposure as the main variables to predict hearing thresholds at frequencies of 3, 4 and 6 kHz. The results showed that these variables contributed 62.1% of dependent variables (*R*^*2*^ = 0.39).

However, several studies suggested that the complex structures or inter-correlation of variables during the development of LR model resulted in the neglect of relation and cross-validation shrinkage (Abdollahi et al. 2018; Bing et al. 2018; Zhang et al. 2016). Consequently, the distinct characteristics of two types of models should be considered and developed to achieve the better performance of each kind of models. For instance, it is more efficient to apply regression models to determine the effect of a specific factor (e.g. type of noise) on the generation of NIHL, because of the greater requirements of ML to produce a similar performance. Concurrently, due to the nature of black box, the exact relationship between inputs and outputs is hard to interpret from ML models (Castelvecchi 2016), which may prevent researchers from focusing on the specific factors that cause high risk of hearing impairment. By contrast, if a study aims to construct an ML model to predict a type of hearing loss with specific etiology, comprehensive variables should be extracted and evaluated before training the model to promise the complexity/flexibility.

Although several algorithms showed a favorable predictive ability, either for NIHL or SNHL with specific etiologies, RF and SVM were one of the most frequently adopted models and are highly recommended for classifying or identifying hearing loss. Random forest is characterized by a combination of decision tree predictors, from which the most voted class is selected to represent the final prediction (Breiman 2001). It is fast to classify, insusceptible to noise, and does not overfit (Singh et al. 2016). On the other hand, as a linear machine learning model SVM could handle both the regression and classification problem with the manual selection of data set (Bing et al. 2018). It usually reaches high accuracy and is tolerant to unrelated features as well as favorable to generalize (Singh et al. 2016). SVM is also able to change to non-linear when it applies the kernel function in the training phase (Kotsiantis et al. 2007), which is more commonly used to predict hearing-related problems. Notably, because the limited data size, recent deep learning techniques have not been used properly in this field yet, such as deep neural network (DNN). Hung et al. (2017) supported that DNN outperformed than LR and SVM in predicting the occurrence of 5-year stroke. With more data, deep neural networks are expected to perform better than the other ML techniques.

Furthermore, it should be noted that due to the difference of designs and datasets between individual studies, multiple algorithm use should be encouraged to examine which model performs best with specific types of data. For instance, two papers suggested that either RF (Statnikov et al. 2008) or SVM (Statnikov and Aliferis 2007) could outperform any other in classification accuracy to diagnose and predict a similar clinical problem. Similarly, Bing et al. (2018) found that deep belief network reached highest performance to predict SSNHL measured by several metrics, whereas SVM was the best classifier compared to predict unilateral SSNHL in another study (Park et al. 2020).

### Limitation of ML models to predict NIHL

One major limitation discovered in the majority of the included studies was that the discrimination risk of the prediction model was seldom evaluated. Two studies reported the ROC curves and only one study evaluated the AUC in the included studies (Zhao et al. 2019a). Similarly, the ROC/AUC was merely estimated in 4 of 9 studies that predicted other types of hearing loss (Acır et al. 2006; Bing et al. 2018; Park et al. 2020; Ziavra et al. 2004), ranging from 0.73 to 0.94. The ROC curve sheds light on the power of a model to discriminate different groups, reflected by the values of true positive rate (sensitivity) and false positive rate (1-specificity). Notably, the superiority of sensitivity and specificity could be different when facing specific predicting problems (Obuchowski and Bullen 2018). In particular, considering the negative consequence and irreversible impact of hearing loss a lower false positive rate is much more important than increasing true positive rate.

Furthermore, feature selection may influence the quality of ML algorithms at the same time. Although the included studies considered variables related to the generation of NIHL, those symptoms that share the similar pathology of NIHL may act as predictors as well. For example, 20–67% of subjects with NIHL showed audiometric ‘notches’ in different studies (Hsu et al. 2013; Lie et al. 2015; Rabinowitz et al. 2006), whereas tinnitus is the primary symptom in some cases without having any audiometric ‘notches’ or hearing loss (Mrena et al. 2007). It should be noted that overfitting would be expected when applying new data to the model, if idiosyncratic features are not eliminated before the training phase (Moons et al. 2014). Several studies discovered that including less relevant variables would undermine the performance of ML models (Bing et al. 2018; Park et al. 2020). On the other hand, the way to process data is important for model prediction. Dichotomizing data usually increases the risk of bias, especially for those around both sides of cut-off points. Compared with continuous and category variables, simply dividing data into two categories, even if it is based on recommendations from other studies, may reduce the information in the data and lower the predictability and applicability, which may eventually give rise to overfitting problem (Moons et al. 2014).

Although the risk of bias, which is more likely to increase when the data set is small, was considered and calibrated in the included studies by two internal validation methods (split-sample validation and n-fold cross-validation), Vabalas et al. (2019) argued that N-fold cross-validation still produced biased prediction with samples less than 1000, whereas split-sample validation achieved better performance in the smaller size data sets. On the other hand, although no studies utilized external validation, which refers to testing ML models using new data or a separate dataset, external validation is more reliable to validate ML models and to help recalibrate the model, therefore is highly recommended (Moons et al. 2014; Vabalas et al. 2019), due to its temporal or spatial difference from the initial datasets compared with internal validation. Apart from the method of validation, separating training and testing data before the model development is imperative to prevent overfitting, because the model is pruned to perform better in the data set where it is derived (Austin and Steyerberg 2017).

### Recommendations of ML models to predict NIHL

According to the limitations found in the included studies, the following recommendations are proposed to maximum the transparency and reproducibility of future studies. First, report all details of steps during model construction, including data collection, feature extraction and selection, model development and model evaluation. We highly recommend to follow the TRIPOD checklist (Moons et al. 2015) to lower the potential risk of bias. Second, recruit more relevant predictors that are correlated with noise-induced hearing loss and evaluate the statistical significance of inputs to prevent overfitting before the training phase. Furthermore, select appropriate validation methods based on sample size. If the number of participants is less than 1,000, split-sample validation should be considered at first, otherwise apply n-fold cross-validation. If possible, external validation is preferable to better evaluate the generalization of models. Finally, it is necessary to analyze more metrics other than accuracy to assess calibration (e.g. precision, recall, prediction error) and discrimination performance (the ROC/AUC curve). The predictive results of both training and testing phases should be separated and informed to eliminate the risk of bias.

The main limitation of the current review is the limited number of included studies and number of algorithms, which might not provide  robust  evidence to represent the performance of machine learning models in predicting NIHL. Furthermore, the heterogeneity of methodology and evaluation methods rendered it more difficult to evaluate and compare the quality of individual prediction models. The effects of different factors on the performance during model development were not analyzed, such as sample size, the number of variables or the number of events per variable.

Further research is expected to recruit more participants and include more predictors relevant to noise-induced hearing loss (e.g. genes, cellular biomarkers) or noise exposure (e.g. the waveform of ABR and OAE) to explore the pathology of noise-induced hearing loss or noise-induced hearing problem, such as hidden hearing loss, noise-induced tinnitus and hyperacusis. In addition, with larger sample sizes by sharing the collected data with each other, better and more powerful ML techniques (e.g. deep learning) could be successfully applied in this field.

## Conclusion

Eight studies were reviewed in the current study and supported relatively high accuracy and/or low prediction error of machine learning in predicting noise-induced hearing impairment. However, limited studies evaluated the discrimination risk of the prediction models and disappointing sensitivity and specificity values were observed from the ROC curves. The above findings revealed several issues when developing ML models, which mainly comprised limited sample sizes, single algorithm use, incomplete reports of model construction, and/or insufficient evaluation of calibration and discrimination. Application of machine learning models or traditional regression models should be based on aims and designs of their studies. Future study would be expected to have bigger sample sizes and increased numbers of predictors relevant to noise-induced hearing loss or noise exposure.

## References

[CR1] Abdollahi H, Mostafaei S, Cheraghi S, Shiri I, Rabi Mahdavi S, Kazemnejad A (2018). Cochlea CT radiomics predicts chemoradiotherapy induced sensorineural hearing loss in head and neck cancer patients: a machine learning and multi-variable modelling study. Phys Med.

[CR2] Acır N, Özdamar Ö, Güzeliş C (2006). Automatic classification of auditory brainstem responses using SVM-based feature selection algorithm for threshold detection. Eng Appl Artif Intell.

[CR3] Alba AC (2017). Discrimination and calibration of clinical prediction models: users’ guides to the medical literature. JAMA.

[CR4] Aliabadi M, Farhadian M, Darvishi E (2015). Prediction of hearing loss among the noise-exposed workers in a steel factory using artificial intelligence approach. Int Arch Occup Environ Health.

[CR5] Alin A (2010). Multicollinearity WIREs. Comput Statist.

[CR6] Altman DG, Royston P (2006). The cost of dichotomising continuous variables. BMJ.

[CR7] Arenas JP, Suter AH (2014). Comparison of occupational noise legislation in the Americas: an overview and analysis. Noise Health.

[CR8] Austin PC, Steyerberg EW (2017). Events per variable (EPV) and the relative performance of different strategies for estimating the out-of-sample validity of logistic regression models. Stat Methods Med Res.

[CR9] Basner M, Babisch W, Davis A, Brink M, Clark C, Janssen S, Stansfeld S (2014). Auditory and non-auditory effects of noise on health. Lancet.

[CR10] Bing D (2018). Predicting the hearing outcome in sudden sensorineural hearing loss via machine learning models. Clin Otolaryngol.

[CR11] Bovo R, Ciorba A, Martini A (2007). Genetic factors in noise induced hearing loss. Audiological Medicine.

[CR12] Breiman L (2001). Random forests. Mach Learn.

[CR13] Castelvecchi D (2016). Can we open the black box of AI?. Nature News.

[CR14] Chang Y-S, Park H, Hong SH, Chung W-H, Cho Y-S, Moon IJ (2019). Predicting cochlear dead regions in patients with hearing loss through a machine learning-based approach: a preliminary study. PLoS ONE.

[CR15] Christodoulou E, Ma J, Collins GS, Steyerberg EW, Verbakel JY, Van Calster B (2019). A systematic review shows no performance benefit of machine learning over logistic regression for clinical prediction models. J Clin Epidemiol.

[CR16] de Waal R, Hugo R, Soer M, Krüger JJ (2002). Predicting hearing loss from otoacoustic emissions using an artificial neural network S Afr. J Commun Disord.

[CR17] Deafness WHOPftPo Hearing I (1998). Prevention of noise-induced hearing loss: report of an informal consultation held at the World Health Organization, Geneva, on 28–30 October 1997.

[CR18] ElahiShirvan H, Ghotbi-Ravandi M, Zare S, Ahsaee M (2020). Using audiometric data to weigh and prioritize factors that affect workers’ hearing loss through support vector machine (SVM). Algorithm Sound Vibrat.

[CR19] Fabris F, De Magalhães JP, Freitas AA (2017). A review of supervised machine learning applied to ageing research. Biogerontology.

[CR20] Farhadian M, Aliabadi M, Darvishi E (2015). Empirical estimation of the grades of hearing impairment among industrial workers based on new artificial neural networks and classical regression methods Indian. J Occup Environ Med.

[CR21] Fligor BJ, Cox LC (2004). Output levels of commercially available portable compact disc players and the potential risk to hearing. Ear Hear.

[CR22] Greenwell BM, Tvaryanas AP, Maupin GM (2018). Risk factors for hearing decrement among U.S Air force aviation-related personnel. Aerosp Med Hum Perform.

[CR23] Hirose K, Liberman MC (2003). Lateral wall histopathology and endocochlear potential in the noise-damaged mouse cochlea. J Assoc Res Otolaryngol.

[CR24] Hsu T-Y, Wu C-C, Chang J-G, Lee S-Y, Hsu C-J (2013). Determinants of bilateral audiometric notches in noise-induced hearing loss. Laryngoscope.

[CR25] Hung C-Y, Chen W-C, Lai P-T, Lin C-H, Lee C-C (2017) Comparing deep neural network and other machine learning algorithms for stroke prediction in a large-scale population-based electronic medical claims database. In: 2017 39th annual international conference of the IEEE engineering in medicine and biology society (EMBC), IEEE 3110–3113 10.1109/EMBC.2017.8037515 29060556

[CR26] Hyndman RJ, Koehler AB (2006). Another look at measures of forecast accuracy. Int J Forecast.

[CR27] Imam L, Hannan SA (2017). Noise-induced hearing loss: a modern epidemic?. Br J Hosp Med (Lond).

[CR28] Jansen EJM, Helleman HW, Dreschler WA, de Laat JAPM (2009). Noise induced hearing loss and other hearing complaints among musicians of symphony orchestras. Int Arch Occup Environ Health.

[CR29] Kähäri KR, Axelsson A, Hellström PA, Zachau G (2001). Hearing development in classical orchestral musicians. A follow-up study Scand Audiol.

[CR30] Kim YS, Cho YH, Kwon OJ, Choi SW, Rhee KY (2011). The risk rating system for noise-induced hearing loss in korean manufacturing sites based on the 2009 survey on work environments. Saf Health Work.

[CR31] Konings A (2007). Association between variations in CAT and noise-induced hearing loss in two independent noise-exposed populations. Hum Mol Genet.

[CR32] Korver AMH (2017). Congenital hearing loss. Nat Rev Dis Primers.

[CR33] Kotsiantis SB, Zaharakis I, Pintelas P (2007). Supervised machine learning: a review of classification techniques. Emerg Artific Intell Appl Comp Eng.

[CR34] Krawczyk B (2016). Learning from imbalanced data: open challenges and future directions progress in artificial. Intelligence.

[CR35] Kuncheva LI (2014) Combining pattern classifiers: methods and algorithms. John Wiley & Sons

[CR36] Lever J, Krzywinski M, Altman N (2016) Points of Significance: Model selection and overfitting. Nature methods 13(9):703-704. 10.1038/nmeth.3968

[CR37] Liberman MC (2016). Noise-induced hearing loss: permanent versus temporary threshold shifts and the effects of hair cell versus neuronal degeneration. Adv Exp Med Biol.

[CR38] Lie A, Skogstad M, Johnsen TS, Engdahl B, Tambs K (2015). The prevalence of notched audiograms in a cross-sectional study of 12,055 railway workers. Ear Hear.

[CR39] Lie A (2016). Occupational noise exposure and hearing: a systematic review. Int Arch Occup Environ Health.

[CR40] Liu Y-W, Kao S-L, Wu H-T, Liu T-C, Fang T-Y, Wang P-C (2020). Transient-evoked otoacoustic emission signals predicting outcomes of acute sensorineural hearing loss in patients with Ménière's disease. Acta Otolaryngol.

[CR41] Low DM, Bentley KH, Ghosh SS (2020). Automated assessment of psychiatric disorders using speech: a systematic review Laryngoscope Investigative. Otolaryngology.

[CR42] McKearney RM, MacKinnon RC (2019). Objective auditory brainstem response classification using machine learning. Int J Audiol.

[CR43] Mellor JC, Stone MA, Keane J (2018). Application of data mining to “big data” acquired in audiology: principles and potential. Trend Hear.

[CR44] Meyer-Bisch C (1996). Epidemiological evaluation of hearing damage related to strongly amplified music (personal cassette players, discotheques, rock concerts)–high-definition audiometric survey on 1364 subjects. Audiology.

[CR45] Mohd Nawi N, Rehman Gillani SM, Ghazali MI (2011). Noise-induced hearing loss prediction in Malaysian industrial workers using gradient descent with adaptive momentum algorithm. Int Rev Comp Software.

[CR46] Molina ME, Perez A, Valente JP (2016). Classification of auditory brainstem responses through symbolic pattern discovery. Artif Intell Med.

[CR47] Moons KGM (2014). Critical appraisal and data extraction for systematic reviews of prediction modelling studies: the CHARMS checklist. PLoS Med.

[CR48] Moons KGM (2015). Transparent reporting of a multivariable prediction model for individual prognosis or diagnosis (TRIPOD): explanation and elaboration. Ann Intern Med.

[CR49] Moshammer H, Kundi M, Wallner P, Herbst A, Feuerstein A, Hutter H-P (2015). Early prognosis of noise-induced hearing loss. Occup Environ Med.

[CR50] Mrena R, Ylikoski M, Mäkitie A, Pirvola U, Ylikoski J (2007). Occupational noise-induced hearing loss reports and tinnitus in Finland. Acta Otolaryngol.

[CR51] Nelson DI, Nelson RY, Concha-Barrientos M, Fingerhut M (2005). The global burden of occupational noise-induced hearing loss. Am J Ind Med.

[CR52] Obuchowski NA, Bullen JA (2018). Receiver operating characteristic (ROC) curves: review of methods with applications in diagnostic medicine. Phys Med Biol.

[CR53] Opperman DA, Reifman W, Schlauch R, Levine S (2006). Incidence of spontaneous hearing threshold shifts during modern concert performances. Otolaryngol Head Neck Surg.

[CR54] Park KV, Oh KH, Jeong YJ, Rhee J, Han MS, Han SW, Choi J (2020). Machine learning models for predicting hearing prognosis in unilateral idiopathic sudden sensorineural hearing loss. Clin Exp Otorhinolaryngol.

[CR55] Pawelczyk M (2009). Analysis of gene polymorphisms associated with K ion circulation in the inner ear of patients susceptible and resistant to noise-induced hearing loss. Ann Hum Genet.

[CR56] Rabinowitz PM (2000). Noise-induced hearing loss. Am Fam Physician.

[CR57] Rabinowitz PM, Galusha D, Slade MD, Dixon-Ernst C, Sircar KD, Dobie RA (2006). Audiogram notches in noise-exposed workers. Ear Hear.

[CR58] Roberts B, Seixas NS, Mukherjee B, Neitzel RL (2018). Evaluating the risk of noise-induced hearing loss using different noise measurement criteria. Ann Work Expo Health.

[CR59] Ryan AF, Kujawa SG, Hammill T, Le Prell C, Kil J (2016). Temporary and permanent noise-induced threshold shifts: a review of basic and clinical observations. Otol Neurotol.

[CR60] Sayler SK, Rabinowitz PM, Galusha D, Sun K, Neitzel RL (2019). Hearing protector attenuation and noise exposure among metal manufacturing workers. Ear Hear.

[CR61] Siblini W, Fréry J, He-Guelton L, Oblé F, Wang Y-Q (2020) Master your metrics with calibration. In: international symposium on intelligent data analysis, Springer 457–469

[CR62] Singh A, Thakur N, Sharma A (2016) A review of supervised machine learning algorithms. In: 2016 3rd International conference on computing for sustainable global development (INDIACom), Ieee, 1310–1315

[CR63] Singhi SK, Liu H (2006) Feature subset selection bias for classification learning. In: proceedings of the 23rd international conference on Machine learning 849–856

[CR64] South T (2013) Managing noise and vibration at work, Routledge

[CR65] Statnikov A, Aliferis CF (2007). Are random forests better than support vector machines for microarray-based cancer classification?. AMIA Annu Symp Proc.

[CR66] Statnikov A, Wang L, Aliferis CF (2008). A comprehensive comparison of random forests and support vector machines for microarray-based cancer classification. BMC Bioinform.

[CR67] Tharwat A (2020). Classification assessment methods. Appl Comput Inf.

[CR68] Tikka C, Verbeek JH, Kateman E, Morata TC, Dreschler WA, Ferrite S (2017). Interventions to prevent occupational noise-induced hearing loss. Cochrane Database Syst Rev.

[CR69] Tomiazzi JS, Pereira DR, Judai MA, Antunes PA, Favareto APA (2019). Performance of machine-learning algorithms to pattern recognition and classification of hearing impairment in Brazilian farmers exposed to pesticide and/or cigarette smoke. Environ Sci Pollut Res Int.

[CR70] Vabalas A, Gowen E, Poliakoff E, Casson AJ (2019). Machine learning algorithm validation with a limited sample size. PLoS ONE.

[CR71] Van Laer L (2006). The contribution of genes involved in potassium-recycling in the inner ear to noise-induced hearing loss. Hum Mutat.

[CR72] Williams W, Brumby S, Calvano A, Hatherell T, Mason H, Mercer-Grant C, Hogan A (2015). Farmers' work-day noise exposure. Aust J Rural Health.

[CR73] Wong ACY, Froud KE, Hsieh YS-Y (2013). Noise-induced hearing loss in the 21st century: a research and translational update. World J Otorhinolaryngol.

[CR74] Xie H-W, Qiu W, Heyer NJ, Zhang M-B, Zhang P, Zhao Y-M, Hamernik RP (2016). The use of the kurtosis-adjusted cumulative noise exposure metric in evaluating the hearing loss risk for complex noise. Ear Hear.

[CR75] Zare S, Hasheminejad N, Shirvan HE, Hasanvand D, Hemmatjo R, Ahmadi S (2018). Assessing individual and environmental sound pressure level and sound mapping in Iranian safety shoes factory. Roman J Acoust Vibrat.

[CR76] Zare S, Ghotbi-Ravandi MR, ElahiShirvan H, Ahsaee MG, Rostami M (2019). Predicting and weighting the factors affecting workers' hearing loss based on audiometric data using C5 algorithm. Ann Glob Health.

[CR77] Zare S, Ghotbiravandi MR, Elahishirvan H, Ahsaeed MG, Rostami M, Esmaeili R (2020). Modeling and predicting the changes in hearing loss of workers with the use of a neural network data mining algorithm. Field Study.

[CR78] Zhang X, Yuan Z, Ji J, Li H, Xue F (2016). Network or regression-based methods for disease discrimination: a comparison study. BMC Med Res Methodol.

[CR79] Zhao Y, Li J, Zhang M, Lu Y, Xie H, Tian Y, Qiu W (2019). Machine learning models for the hearing impairment prediction in workers exposed to complex industrial noise: a pilot study. Ear Hear.

[CR80] Zhao Y, Tian Y, Zhang M, Li J, Qiu W (2019). Development of an automatic classifier for the prediction of hearing impairment from industrial noise exposure. J Acoust Soc Am.

[CR81] Ziavra N, Kastanioudakis I, Trikalinos TA, Skevas A, Ioannidis JPA (2004). Diagnosis of sensorineural hearing loss with neural networks versus logistic regression modeling of distortion product otoacoustic emissions. Audiol Neurootol.

